# New Strategies for Treatment of Sepsis

**DOI:** 10.3390/medicina56100527

**Published:** 2020-10-10

**Authors:** Antonio Mirijello, Alberto Tosoni, on behalf of the Internal Medicine Sepsis Study Group

**Affiliations:** 1 Internal Medicine Unit, Department of Medical Sciences, IRCCS Casa Sollievo della Sofferenza, 71013 San Giovanni Rotondo, Italy; 2 Department of Internal Medicine and Gastroenterology, Fondazione Policlinico Universitario “A. Gemelli” IRCCS, 00168 Rome, Italy

**Keywords:** internal medicine, intensive care, emergency department, organ dysfunction, immunomodulation, micronutrients, antimicrobial stewardship

## Abstract

Sepsis represents a major global health concern and is one of the most feared complications for hospitalized patients, being the cause, directly or indirectly, of about half of all hospital deaths. According to the last definition, sepsis is a life-threatening organ dysfunction caused by a dysregulated host response to infection and defined septic shock as a subset of sepsis in which underlying circulatory and cellular/metabolic abnormalities are profound enough to significantly increase mortality. Sepsis is a time-dependent disease and requires a prompt recognition and a standardized treatment. The Special Issue “New Strategies for Treatment of Sepsis” has been thought to connect the experience of physicians involved in the diagnosis, management, and treatment of sepsis at every stage of disease, from emergency departments to general and intensive wards. The focus will be pointed on new approaches to this syndrome, such as early recognition based on clinical features and biomarkers, management in non-ICUs, non-invasive treatment strategies, including non-antimicrobial agents, and, of course, invasive approaches. This Special Issue will highlight the many different facets of sepsis, seen through the eyes of different specialists. We hope to spread the knowledge of a new blueprint for treatment.

Sepsis represents a major global health concern [1] and is one of the most feared complications for hospitalized patients, being the cause, directly or indirectly, of about half of all hospital deaths [2].

The definition of sepsis has changed during the years, with progressive attempts to provide a more defined picture of its real nature: a time-dependent syndrome, requiring early recognition and effective treatment. Thus, the last consensus conference defined sepsis as a life-threatening organ dysfunction caused by a dysregulated host response to infection and defined septic shock as a subset of sepsis in which underlying circulatory and cellular/metabolic abnormalities are profound enough to significantly increase mortality [3].

Although, in the last few decades, sepsis was managed quite exclusively by intensivists within intensive care units (ICUs), in recent years, there has been a progressive increase in admissions of septic patients to non-ICU wards, in particular internal medicine wards [4]. This change is effected for several reasons. First, patients have become progressively older and sicker (e.g., affected by multiple chronic diseases), often giving fewer chances to benefit from intensive treatments. Moreover, the early recognition and management of sepsis and septic shock has significantly improved, positively impacting on the prognosis of these patients. As a consequence, there is a growing collection of literature data derived from studies conducted in non-ICU settings, adding useful information for the management of sepsis with less invasive strategies, filling gaps of knowledge for non-intensivists and/or confirming previously acquired know-hows.

Being a time-dependent disease, sepsis requires a prompt recognition and a standardized approach for an optimal treatment. In general medicine wards, the main limitations to this purpose are represented by the absence of classical signs/symptoms of infection (e.g., fever) [5], the unfavorable proportion of patients vs. staff, and an environment with no advanced monitoring tools [4].

At present, there are still several unmet needs that should be addressed. The comprehension of mechanisms underlying the development and progression of sepsis, the use of new diagnostic tools [6] for a better and less invasive approach, including artificial intelligence, and the development of antimicrobial strategies in order to effectively fight antimicrobial resistance represent only a few of these.

On the other hand, returning to the most recent definition of sepsis, it still remains very generic and impractical. An organ dysfunction caused by a dysregulated host response to infection, for example, is a phrase that can well describe even severe forms of COVID-19 [7,8]. In this regard, this is only one of the many faces with which sepsis can manifest itself and is one of the many different pathophysiological mechanisms via which organ failure can develop. This is the reason why one of the objectives of this Special Issue is to carry out personalized medicine in the field of sepsis, based on the ability to identify its different manifesting typologies. Given all the variables involved (site and type of infection, microbial etiology, host comorbidity, genetic predisposition, released cytokines, hospital care setting, etc.), defining a specific, tailor-made treatment remains hard issue, however desirable.

The Special Issue “New Strategies for Treatment of Sepsis” has been thought to connect the experience of physicians involved in the diagnosis, management, and treatment of sepsis at every stage of disease, from emergency departments to general and intensive wards. The focus will be pointed on new approaches to this syndrome, such as early recognition based on clinical features and biomarkers, management in non-ICUs, non-invasive treatment strategies, including non-antimicrobial agents, and, of course, invasive approaches.

This Special Issue will highlight the many different facets of sepsis, seen through the eyes of different specialists. We hope to spread the knowledge of a new blueprint for treatment.

## References

[1] Reinhart K., Daniels R., Kissoon N., Machado F.R., Schachter R.D., Finfer S. (2017). Recognizing sepsis as a global health priority—A WHO resolution. N. Engl. J. Med..

[2] Rudd K.E., Johnson S.C., Agesa K.M., Shackelford K.A., Tsoi D., Kievlan D.R., Colombara D.V., Ikuta K.S., Kissoon N., Finfer S. (2020). Global, regional, and national sepsis incidence and mortality, 1990–2017: Analysis for the Global Burden of Disease Study. Lancet.

[3] Singer M., Deutschman C.S., Seymour C.W., Shankar-Hari M., Annane D., Bauer M., Bellomo R., Bernard G.R., Chiche J.D., Coopersmith C.M. (2016). The third international consensus definitions for sepsis and septic shock (Sepsis-3). JAMA.

[4] Zaccone V., Tosoni A., Passaro G., Vallone C.V., Impagnatiello M., Li Puma D.D., De Cosmo S., Landolfi R., Mirijello A., Internal Medicine Sepsis Study Group (2017). Sepsis in Internal Medicine wards: Current knowledge, uncertainties and new approaches for management optimization. Ann. Med..

[5] Mirijello A., Tosoni A., Zaccone V., Impagnatiello M., Passaro G., Vallone C.V., Cossari A., Ventura G., Gambassi G., De Cosmo S. (2019). MEDS score and vitamin D status are independent predictors of mortality in a cohort of Internal Medicine patients with microbiological identified sepsis. Eur. Rev. Med Pharmacol. Sci..

[6] Tosoni A., Paratore M., Piscitelli P., Addolorato G., De Cosmo S., Mirijello A., Internal Medicine Sepsis Study Group (2020). The use of procalcitonin for the management of sepsis in Internal Medicine wards: Current evidence. Panminerva Med..

[7] Wiersinga W.J., Rhodes A., Cheng A.C., Peacock S.J., Prescott H.C. (2020). Pathophysiology, Transmission, Diagnosis, and Treatment of Coronavirus Disease 2019 (COVID-19): A Review. JAMA.

[8] Lin H.Y. (2020). The severe COVID-19: A sepsis induced by viral infection? And its immunomodulatory therapy. Chin. J. Traumatol..

